# Rocio virus sustained circulation in Brazil: first infection case in a horse highlights the need for enhanced arbovirus surveillance

**DOI:** 10.1007/s00705-026-06574-9

**Published:** 2026-02-28

**Authors:** Tiago Gräf, Maria Constanza Rodriguez, Yara de Oliveira Brandão, Carla Adriane Royer, Caroline do Nascimento Ferreira, Camila Confortin, Camila Zanluca, Daisy Maria Strottmann, Claudia Nunes Duarte dos Santos, Mara Eliza Gasino Joineau

**Affiliations:** 1 Laboratório de Virologia Molecular, Instituto Carlos Chagas, Fundação Oswaldo Cruz, Curitiba, Brazil; 2 Marcos Enrietti Diagnostic Center, Paraná Agricultural Defense Agency, Rua Jaime Balão, 575, 80040-340 Curitiba, Paraná Brazil

## Abstract

Rocio virus (ROCV), a neurotropic arbovirus of the *Orthoflavivirus* genus, caused Brazil’s largest encephalitis outbreak in the 1970s but has since been rarely detected. We report the first ROCV encephalitis clinical case after nearly 40 years and isolated and sequenced the virus from CNS tissue of a horse. Metatranscriptomics enabled full-genome sequencing, revealing divergence from the reference sequence at an evolutionary rate of 5.8–7.0 × 10⁻⁴, consistent with *Orthoflavivirus*. Thirty-one non-synonymous substitutions were identified, with the envelope protein most affected. Findings indicate ROCV cryptic circulation and ongoing evolution. Horses may act as sentinels, underscoring the need for strengthened ROCV genomic surveillance.

**Supplementary Information:**

The online version contains supplementary material available at 10.1007/s00705-026-06574-9.

Rocio virus (ROCV) is an arbovirus of the genus *Orthoflavivirus*, whose circulation was so far only reported in Brazil [1]. ROCV is currently classified as a subspecies of Ilheus virus (ILHV, *Orthoflavivirus ilheusense*) — a South American indigenous arbovirus with a broad circulation in the continent [2]. Both viruses are maintained in enzootic cycles between birds and arboreal mosquitoes in which humans are incidental dead-end hosts. Infection symptoms in humans fit in the dengue-like clinical spectrum, however, progression to encephalitis is much more frequent since both are neurotropic viruses [1].

ROCV was responsible for the largest viral encephalitis outbreak ever reported in Brazil. In the years of 1975, 1976 and 1977, a series of outbreaks of human encephalitis were reported in the coastal region of São Paulo state, affecting several municipalities [3, 4]. In this context, a new virus was isolated from central nervous system (CNS) tissue of a deceased patient in December 1975 and named after the Rocio neighborhood, where the patient lived [5]. Nine further ROCV isolates were then obtained from CNS of encephalitis deceased patients in 1976, establishing the causal correlation between the virus and the outbreak. During the three years of outbreaks more than 1000 encephalitis cases were reported in the region with a case fatality rate above 10% and permanent neurologic sequelae above 20% [1].

After the 1970’s outbreaks, ROCV has surprisingly disappeared and only few additional positive individuals were found in serosurveys across Brazil [1]. The only two cases detected by molecular biology and sequencing methods were reported in the state of Goiás in 2012 and 2013, after screening samples with dengue-like symptoms [6]. Interestingly, a serosurvey study in horses sampled in five different Brazilian states found 6.1% of ROCV monotypic seropositive, indicating that these animals are also susceptible to infection, however, the up to date lack of epizootic reports in horses suggested that these infections were asymptomatic or subclinical [7]. Here, we report the first clinical case of ROCV infection in a horse, from which the virus was isolated, and its complete genome was sequenced and analyzed.

In February 2013, a 5-year-old Quarter Horse mare presenting with neurological symptoms — including hindlimb paralysis, opisthotonus, forelimb tonic-clonic seizures, and self-mutilation — was found in the municipality of Piraquara, Paraná state, Brazil. The horse was euthanized, and CNS tissues were collected and submitted to the Marcos Enrietti Diagnostic Center (CDME), Agência de Defesa Agropecuária do Paraná (ADAPAR), for rabies testing.

Diagnostic procedures included the Fluorescent Antibody Test (FAT) for rabies and Mouse Inoculation Test (MIT) on samples from the cortex, brain stem, thalamus, and cerebellum. Virus isolation was attempted using Madin Darby bovine kidney (MDBK), Baby Hamster Kidney (BHK21), African green monkey kidney (VERO), and Rabbit Kidney Epithelial (RK13) cells. After observing cytopathic effects, a genus-specific RT-PCR for Orthoflavivirus [8] was conducted using cell culture supernatant, and the resulting 220 bp amplicon was sequenced by Sanger method.

To sequence the entire viral genome, the isolate (designated EQ773/13) was submitted to the Laboratório Referência de Vírus Emergentes, Fiocruz Paraná, for metatranscriptomic sequencing. Library preparation was done with the Illumina Stranded Total RNA Prep kit (Illumina), and sequencing was performed on the Illumina MiSeq platform using the MiSeq Reagent Micro V2 kit with a 2 x 151 bp configuration. Sequence reads were trimmed using Trimmomatic [9], and de novo assembly was carried out with SPAdes v4.0 using the metagenomic RNA virus mode [10].

Initial phylogenetic analyses were performed to position the new virus genome in the whole *Orthoflavivirus* genus tree. All complete genomes from exemplar isolates of the *Orthoflavivirus* species recognized by the International Committee on Taxonomy of Viruses (ICTV) were retrieved from GenBank. Sequences were then trimmed to the coding region in Aliview [11], translated to amino acid, aligned in mafft [12] with the L-INS-i strategy and a maximum likelihood (ML) tree was inferred using IQ-TREE [13]. To better understand ROCV evolution, all sequences available in GenBank — from six previously reported ROCV cases (three from the 1970s outbreaks and three from Goiás state, 2012–2013) — were retrieved (Supplementary Table 1). Separate alignments of partial NS5, envelope, and full-genome sequences were generated. ML trees were inferred using IQ-TREE, and temporal signal and evolutionary rate analyses were conducted using Tempest [14] via regression analysis between sampling year and divergence from root-to-tip. To further assess genomic divergence, the EQ773/13 genome was manually annotated based on polyprotein cleavage sites reported for ILHV and Saint Louis encephalitis virus (SLEV) [15]. Non-synonymous mutations were identified by comparison with the ROCV reference genome NC_040776.

FAT results for the horse CNS tissue were negative for rabies, while MIT was MIT was considered inconclusive since the inoculated mice died in the first four days after inoculation, before the 21 days of rabies incubation period. FAT in the mice CNS also yielded negative results. Cytopathic effects were observed in BHK21, VERO, and RK13 cell cultures. Subsequent *Orthoflavivirus* genus-specific RT-PCR and the Sanger sequencing of the 220bp amplicon identified ROCV in the cell culture supernatants.

Metatranscriptomic sequencing and de novo assembly of the full viral genome yielded a 10,849 nt contig with an average k-mer coverage depth of 5,592 and 97.3% nucleotide identity to the ROCV reference sequence (NC_040776). The complete genome of isolate EQ773/13 is available under GenBank accession number PV448280.

The whole *Orthoflavivirus* genus phylogenetic tree revealed that EQ773/13 clustered with high support with the ROCV reference genome and with ILHV (Fig. 1). Both viruses also grouped in a bigger cluster with other important neurotropic viruses such as Saint Louis Encephalitis (SLEV), West Nile (WNV) and Japanese Encephalitis (JEV).

**Fig. 1:**
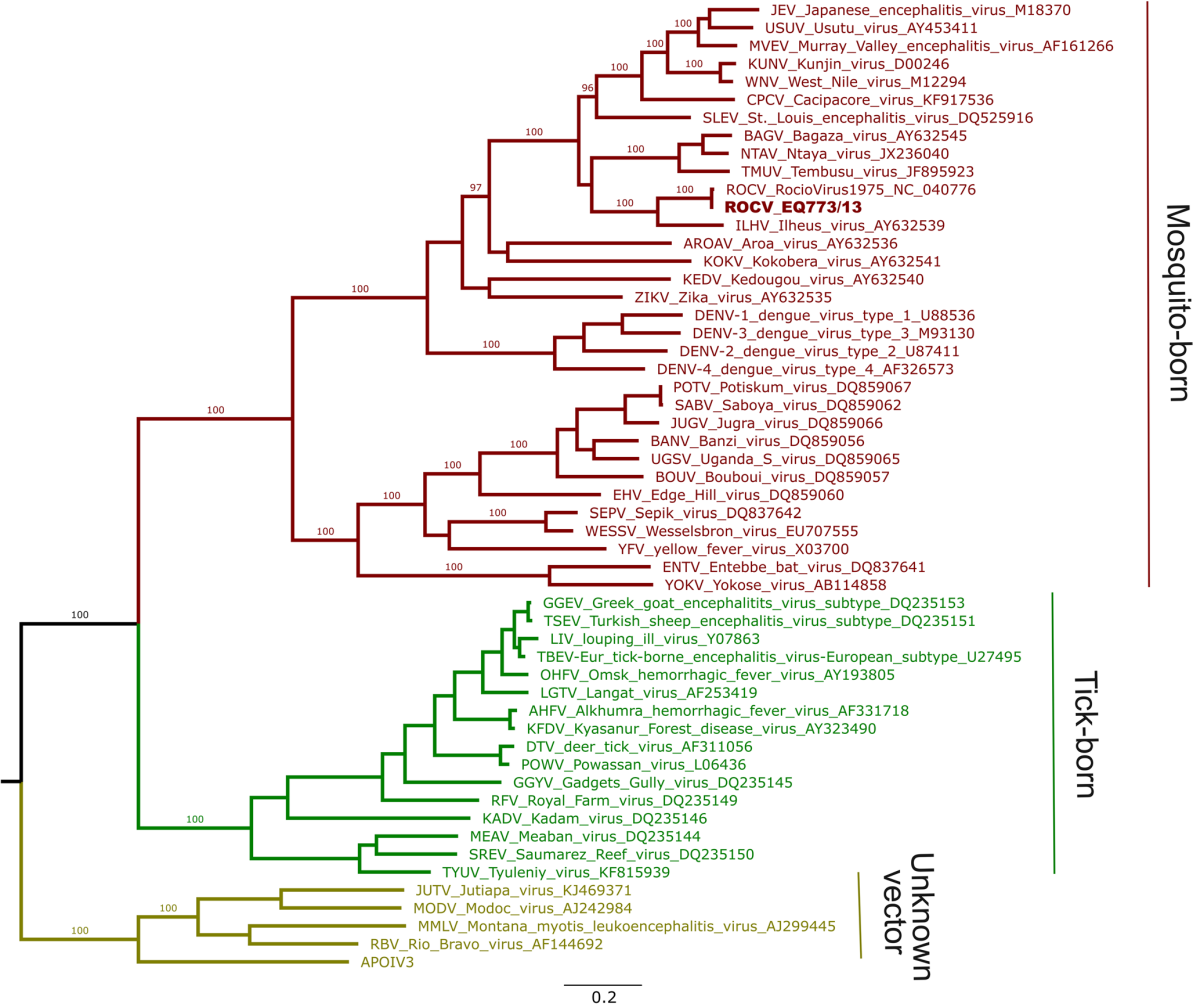
*Orthoflavivirus* genus phylogenetic tree. Whole coding sequences (amino acid translated) from all exemplar isolates of ICTV recognized *Orthoflavivirus* species were included in the tree. ROCV (NC_040776) which is an additional isolate from ILHV species was also included. Branches are colored according to the virus arthropod vector and bootstrap support is shown for key branches

Due to the considerable genetic distance (297 nucleotide mutations) accumulated between EQ773/13 and the 1975 reference strain, we performed a more detailed analysis to infer whether this rate of mutation accumulation was biologically feasible. To this end, we assumed a strict molecular clock, represented by the linear regression analysis line where the slope of the line is the evolutionary rate of the virus (Fig. 2). Despite the very few ROCV sequences available and which were collected basically at two points in time, the estimates from this simple model were consistent with known evolutionary rates for other Orthoflavivirus members such as dengue [16] and WNV [17]. As expected, a faster evolution was observed in the envelope gene compared to NS5, and the root time — inferred by the X-intercept value in the regression analysis — for the whole ROCV phylogenetic tree was estimated in 1972–1973, just two years before the encephalitis outbreaks onset. Important to note that the only three other virus sequences generated after the encephalitis outbreaks don’t follow a clock-like evolution and are unexpectedly similar to the 1970s virus (Fig. 2c). When removing these sequences from the analysis, the inferred evolutionary rate for NS5 aligns with the observed values in other Orthoflavivirus (Fig. 2d).

**Fig. 2:**
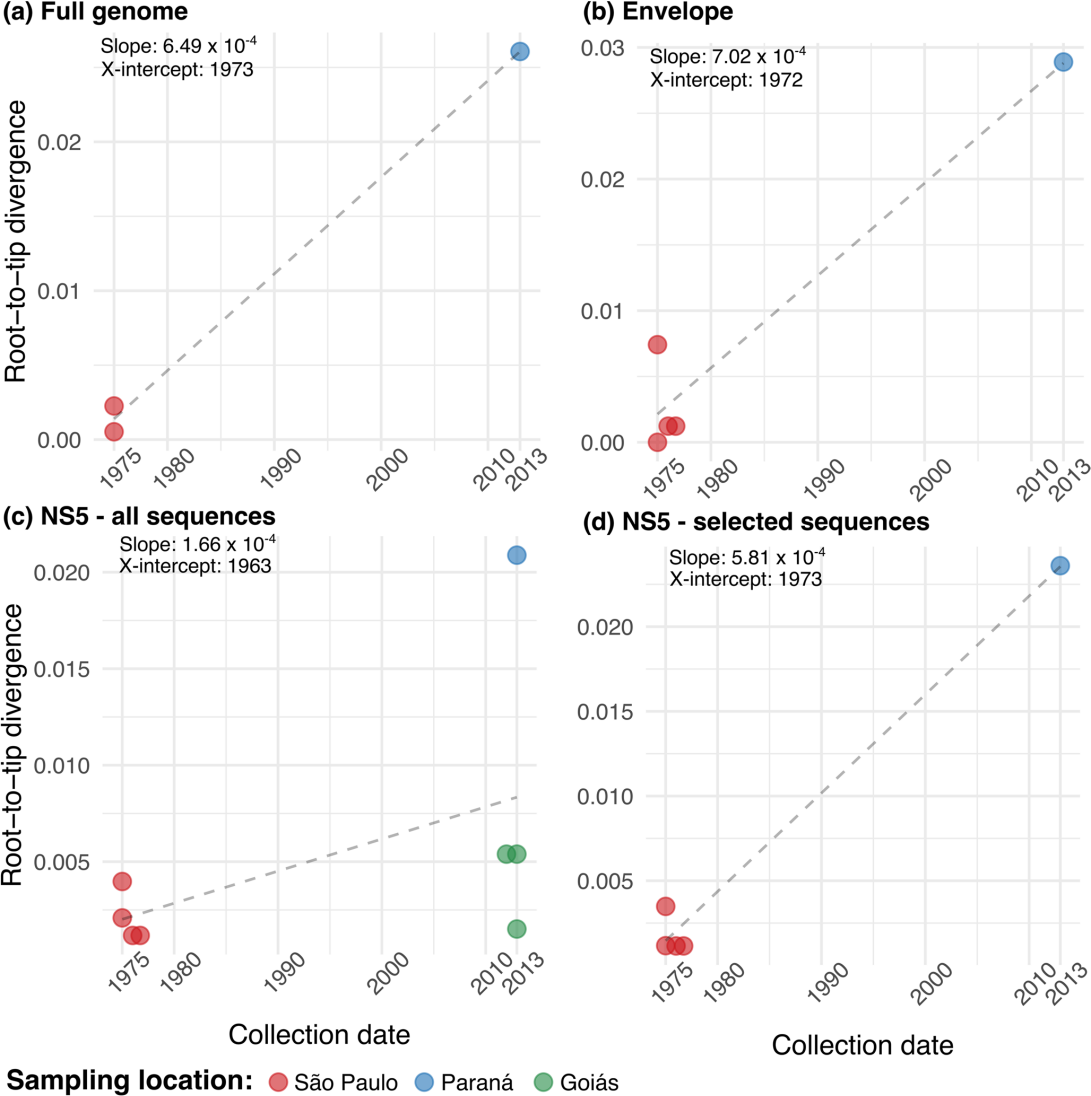
ROCV genetic divergence between 1970’s outbreak sequences and 2013 genome. A regression analysis between the sampling year and the divergence from root-to-tip was inferred from phylogenetic trees with virus full genomes (**a**), envelope sequences (**b**), all available NS5 sequences (**c**) and selected NS5 sequences (**d**). The slope parameter represents ROCV evolutionary rate and X-intercept parameter represents the tree root time

Comparative analysis of the EQ773/13 genome identified 31 amino acid substitutions, with the envelope protein showing the highest mutation rate per site (0.018), including nine unique mutations relative to the NC_040776 reference genome (Table 1).

**Table 1:** ROCV EQ773/13 isolate non-synonymous changes compared to the NC_040776 reference genome

Region	Size (aa)	Total number of changes	Changes per site	Positions
capsid	118	1	0.008	A92V
prM	167	0	0.000	-
E	501	9	0.018	A131E, A132S, T138A, Y216N, V228A, E242A, I245T, V246A, N372S
NS1	353	4	0.011	H16Y, D155E, V164I, R304K
NS2A	227	1	0.004	M107V
NS2B	131	1	0.008	D129E
NS3	619	4	0.006	H139S, S206T, N417D, I471T
NS4A	126	0	0.000	-
2K	23	0	0.000	-
NS4B	256	2	0.008	R8H, S56A
NS5	904	9	0.010	A52V, L175M, F416L, T563M, D622E, T641S, P642R, I676V, K895E

Our results reveal a sustained circulation of ROCV in Brazil, probably in still uncovered animal reservoirs. The observed genetic divergence reflects the virus long term evolution and is in agreement with the genus molecular clock. This is the first case of ROCV infection reported in a horse, highlighting the importance of this animal as a sentinel for the virus reemergence. After nearly four decades, ROCV remains an elusive pathogen, however, the findings of this study serve as a critical warning regarding its potential reemergence. These results underscore the necessity of incorporating ROCV into laboratory surveillance panels to facilitate early outbreak detection and enable timely implementation of containment measures.

## Electronic Supplementary Material

Below is the link to the electronic supplementary material

Supplementary Material 1

## Data Availability

The viral genome generated in this work is available in GenBank under accession number PV448280.
