# Supplementary material for: Rocio virus sustained circulation in Brazil: first infection case in a horse highlights the need for enhanced arbovirus surveillance
Source: Arch Virol. 2026 Feb 28;171(4):106. doi: 10.1007/s00705-026-06574-9 (PMC12948843; doi:10.1007/s00705-026-06574-9)
Supplement: Supplementary file 1 — Supplementary Material 1 [file 705_2026_6574_MOESM1_ESM.docx]

| Accession | Organism Name | GenBank Title | Length | Geo Location | Country | Host | Collection Date | | Release Date | Pub Title | | Pub Link |
| --- | --- | --- | --- | --- | --- | --- | --- | --- | --- | --- | --- | --- |
| NC_040776 / AY632542.4 | Rocio virus | Rocio virus strain SPH 34675, complete genome | 10794 |  |  |  | 1975 | 2019-02-12 | | Biological Transmission of Arboviruses: Reexamination of and New Insights into Components, Mechanisms, and Unique Traits as Well as Their Evolutionary Trends | [16223950](https://www.ncbi.nlm.nih.gov/pubmed/16223950) | |
| MT891158 | Rocio virus | Rocio virus isolate 18 nonstructural protein 5 (NS5) gene, partial cds | 779 | Goiania | Brazil | Homo sapiens | 2013 | 2021-11-08 | | Detection of Rocio Virus SPH 34675 during Dengue Epidemics, Brazil, 2011–2013 | [32186498](https://www.ncbi.nlm.nih.gov/pubmed/32186498) | |
| MT891159 | Rocio virus | Rocio virus isolate 21 nonstructural protein 5 (NS5) gene, partial cds | 189 | Goiania | Brazil | Homo sapiens | 2013 | 2021-11-08 | | Detection of Rocio Virus SPH 34675 during Dengue Epidemics, Brazil, 2011–2013 | [32186498](https://www.ncbi.nlm.nih.gov/pubmed/32186498) | |
| MW446949 | Rocio virus | Rocio virus isolate GYN/2022/human NS5 gene, partial cds | 776 | Goiania | Brazil | Homo sapiens | 2012 | 2021-03-29 | | Detection of Rocio Virus SPH 34675 during Dengue Epidemics, Brazil, 2011–2013 | [32186498](https://www.ncbi.nlm.nih.gov/pubmed/32186498) | |
| MF461639 | Rocio virus | Rocio virus strain SPH 34675, complete genome | 10794 |  | Brazil | Homo sapiens | 1975 | 2017-10-04 | | Full genome sequence of Rocio virus reveal substantial variations from the prototype Rocio virus SPH 34675 sequence | [28939977](https://www.ncbi.nlm.nih.gov/pubmed/28939977) | |
| AY739902 | Rocio virus | Rocio virus strain SPAn 37630 envelope (E) gene, partial cds | 816 | Brazil: Ribeira Valley, Sao Paulo State | Brazil |  | 1976-02-28 | 2006-10-01 | | Molecular characterization of two rocio flavivirus strains isolated during the encephalitis epidemic in são paulo state, brazil and the development of a one-step rt-pcr assay for diagnosis | [10.1590/s0036-46652008000200005](https://doi.org/10.1590/s0036-46652008000200005) | |
| AY739903 | Rocio virus | Rocio virus strain SPH 37623 envelope (E) gene, partial cds | 816 | Brazil: Ribeira Valley, Sao Paulo State | Brazil |  | 1976-03-29 | 2006-10-01 | | Molecular characterization of two rocio flavivirus strains isolated during the encephalitis epidemic in são paulo state, brazil and the development of a one-step rt-pcr assay for diagnosis | [10.1590/s0036-46652008000200005](https://doi.org/10.1590/s0036-46652008000200005) | |
| AY739904 | Rocio virus | Rocio virus strain SPAn 37630 NS5 protein (NS5) gene, partial cds | 864 | Brazil: Ribeira Valley, Sao Paulo State | Brazil |  | 1976-02-28 | 2006-10-01 | | Molecular characterization of two rocio flavivirus strains isolated during the encephalitis epidemic in são paulo state, brazil and the development of a one-step rt-pcr assay for diagnosis | [10.1590/s0036-46652008000200005](https://doi.org/10.1590/s0036-46652008000200005) | |
| AY739905 | Rocio virus | Rocio virus strain SPH 37623 NS5 protein (NS5) gene, partial cds | 864 | Brazil: Ribeira Valley, Sao Paulo State | Brazil |  | 1976-03-29 | 2006-10-01 | | Molecular characterization of two rocio flavivirus strains isolated during the encephalitis epidemic in são paulo state, brazil and the development of a one-step rt-pcr assay for diagnosis | [10.1590/s0036-46652008000200005](https://doi.org/10.1590/s0036-46652008000200005) | |

**Supplementary Table 1.** Publicly available Rocio virus (ROCV) sequences used in this study
